# Effects of COVID-19 pandemic on mental health among frontline healthcare workers: A systematic review and meta-analysis

**DOI:** 10.3389/fpsyg.2022.1096857

**Published:** 2023-01-27

**Authors:** Jie Tong, Jie Zhang, Na Zhu, Yu Pei, Weiqing Liu, Wei Yu, Chengping Hu, Xirong Sun

**Affiliations:** Clinical Research Center for Mental Disorders, Shanghai Pudong New Area Mental Health Center, School of Medicine, Tongji University, Shanghai, China

**Keywords:** mental health, frontline healthcare workers, COVID-19, systematic review, meta-analysis

## Abstract

**Background:**

As some countries announced to remove Coronavirus Disease 2019 (COVID-19) border, it indicates that the COVID-19 may have entered its terminal stage. In this COVID-19 pandemic, the mental health of frontline healthcare workers (HCWs) experienced unprecedented challenges. However, the impact of the COVID-19 pandemic on mental health among frontline HCWs lacks a high-quality and long-term systematic review and meta-analysis.

**Methods:**

We conducted a systematic review and meta-analysis according to PRISMA guidelines. The system searches EMBASE, MEDLINE, PsycINFO, Cochrane Library, ScienceNet, and ERIC. Analyze the mental health problems of frontline HCWs in different regions and periods, including insomnia, stress, anxiety and depression. This study was registered in PROSPERO under the number CRD42021253821.

**Results:**

A total of 19 studies on the effects of COVID-19 pandemic on mental health among frontline HCWs were included in this study. The overall prevalence of insomnia was 42.9% (95% CI, 33.9–51.9%, *I*^2^ = 99.0%) extracted from data from 14 cross-sectional studies (*n* = 10 127), 1 cohort study (*n* = 4,804), and 1 randomized controlled trial (RCT; *n* = 482) in 10 countries. The overall prevalence of stress was 53.0% (95% CI, 41.1–64.9%, *I*^2^ = 78.3%) extracted from data from nine cross-sectional studies (*n* = 5,494) and 1 RCT study (*n* = 482) from eight countries. The overall prevalence of anxiety and depression was 43.0% (95% CI, 33.8–52.3%, *I*^2^ = 99.0%) and 44.6% (95% CI, 36.1–53.1%, *I*^2^ = 99.0%) extracted from data from 17 cross-sectional studies (*n* = 11,727), one cohort study (*n* = 4,804), and one RCT study (*n* = 482) from 12 countries. The prevalence of stress and depression was higher in 2020, while the prevalence of insomnia and anxiety was higher in 2021. The prevalence of mental health problems among physicians was higher than that of other frontline HCWs. The prevalence of mental health problems among frontline HCWs is higher in South America and lower in North America.

**Conclusions:**

This systematic review and meta-analysis showed that the COVID-19 pandemic have significant effects on mental health among frontline HCWs. The overall prevalence of insomnia, stress, anxiety and depression among frontline HCWs is high. Therefore, the health policy-makers should pay attention to and respond to the mental health problems of frontline HCWs in the context of public health emergencies.

**Systematic review registration:**

https://www.crd.york.ac.uk/PROSPERO/.

## Background

The Coronavirus Disease 2019 (COVID-19) pandemic has existed since January 2020 and has become a major global health crisis (Chu et al., 2020; Wu et al., 2020; Gottlieb et al., 2021). The mental health of frontline HCWs has encountered unprecedented challenges because of long-term and high load medical care, COVID-19 prevention, nucleic acid detection and vaccination (Wang W. et al., 2020; Dai et al., 2021; Skowronski and De Serres, 2021). Some studies shows that the prevalence of mental health disorders among frontline HCWs during the COVID-19 pandemic is very high, but the prevalence rates in different studies vary greatly (Wasserman et al., 2020; Lotta et al., 2021; Lumley et al., 2021). Insomnia among frontline HCWs varied across studies from 19.7 to 73.7% (Giardino et al., 2020; Wang L. Q. et al., 2020), those of stress varied from 26.8 to 83.1% (Alshekaili et al., 2020; Elkholy et al., 2021), those of anxiety varied from 14.2 to 77.3% (Wang L. Q. et al., 2020; Elkholy et al., 2021), and those of depression varied from 14.3 to 81.0% (Giardino et al., 2020; Lee et al., 2021). Consequently, there are systematic differences in most of what we know about the mental health risks and problems among frontline HCWs.

Although systematic differences in studies have been the subject of frequent inquiry, the differences may be more prominent under the special background of COVID-19 (Salehi et al., 2020). Particularly, the specialty of HCWs, online surveys, severity of COVID-19, or study regions may lead to substantial changes in the results (Böger et al., 2021). Studies also report conflicting findings about whether frontline HCWs' insomnia, stress, anxiety, and depression vary by sex, specialty, region, or other characteristics (Haravuori et al., 2020; Wang L. Q. et al., 2020). However, few studies have synthesized the effects of various factors on the results. Furthermore, the current studies on the effects of COVID-19 pandemic on mental health mainly focuses on the general population, students, or patients with COVID-19 (Kinder and Harvey, 2020; Salari et al., 2020; Deng et al., 2021). There is a lack of high-quality and long-term systematic review and meta-analysis of the effects of the COVID-19 pandemic on the mental health of frontline HCWs.

Although some rapid systematic reviews or meta-analysis during the outbreak of the pandemic also focused on the mental health of frontline HCWs, they lacked high-quality meta-analysis or did not cover the whole process of the COVID-19 pandemic, and could not obtain long-term, more professional evidence-based data (Pappa et al., 2020; Schneider et al., 2022). As some countries announced to remove COVID-19 border, it indicates that the COVID-19 may have entered its terminal stage. In order to better understand the effects of COVID-19 pandemic on the mental health among frontline HCWs in different periods and regions, we conducted a comprehensive systematic review and meta-analysis focused on evaluating the following questions: (1) What is the overall estimated prevalence of insomnia, stress, anxiety, and depression among frontline HCWs during the COVID-19 pandemic? (2) What are the differences of mental health problems among frontline HCWs in different periods and regions during COVID-19? This unified framework can highlight the mental health problems of frontline HCWs in public health emergencies, and provide health policy-makers with strategic information based on evidence-based medicine.

## Methods

### Search strategy and selection criteria

All studies published between January 1, 2019, and December 31, 2021, that reported on the mental health of frontline HCWs affected by the COVID-19 pandemic, such as insomnia, stress, anxiety, and depression, were identified using EMBASE, MEDLINE, PsycINFO, Cochrane Central Register of Controlled Trials, Cochrane Database of Systematic Reviews, Web of Science, and ERIC. Government databases websites, conference proceedings, and medical society websites were also searched (independently performed by J.T. and W.Q.L.). The investigators screened the reference lists of identified articles using the approaches recommended by the Preferred Reporting Items for Systematic Reviews and Meta-Analysis (PRISMA; Moher et al., 2009). Computer-based searches used terms related to the mental health of frontline HCWs during the COVID-19 pandemic (see Table 1). Studies which published in peer-reviewed journals that reported data on frontline HCWs and used a validated method to assess insomnia, stress, anxiety, and depression were also included.

**Table 1:** Search strategy.

**Insomnia**	**Stress**	**Anxiety**	**Depression**	**Frontline healthcare workers**	**COVID-19**	**Others**
1. Insomnia 2. Sleep disorders 3. Sleep problems 4. OR /# 1 –# 3	5. Stress 6. Stress response 7. Stress disorder 8. Acute stress disorder 9. Traumatic stress disorder 10. OR /#5 –#9	11. Anxiety 12. Anxiety disorder 13. Anxious distress 14. OR / #11 –#13	15. Depress 16. Depressed 17. Depression 18. Depressive disorder 19. Major depression 20. Major depressive disorder 21. MDD 22. OR / #15 –#21	23. Doctor 24. Physician 25. Nurse 26. Health Personnel 27. Health care Provider 28. Health worker 29. Healthcare provider 30. Healthcare worker 31. Frontline healthcare workers 32. Healthcare professional 33. Medical staff 34. Medical worker 35. OR / #23 –#34	36. SARS-CoV-2 37. Infection 38. COVID-19 virus disease 39. 2019 novel coronavirus infection 40. 2019-nCoV infection 41. Coronavirus disease 2019 42. 2019-nCoV disease 43. COVID-19 44. OR / #36 –#43	45. Mental health 46. Psychological problems 47. Psychological disorder 48. Prevalence 49. Incidence 50. OR / #45 –#49
Combined search	#4 OR #10 OR #14 OR #22 AND #35 AND #44 AND #50			

Inclusion criteria must be that the study population is frontline healthcare workers in COVID-19 affected countries or areas. Only high-quality studies evaluating the prevalence rates of specific mental health problems such as insomnia, stress, anxiety and depression are eligible for inclusion. However, mental health problems such as post-traumatic stress disorder, psychotic disorder and obsessive-compulsive disorder were excluded because of the limited number of high-quality studies. Additionally, broad terms such as “psychological distress and psychological abnormality” were excluded as they can be difficult to quantify.

### Data extraction

The investigators (J.Z. and W.Y.) independently extracted the following information from each article using a standardized form: study design, country, survey period, specialty, sample size, average age, diagnostic methods, screening instrument outcome, and reported prevalence of insomnia, stress, anxiety, and depression. To eliminate studies involving the same population or multiple identical publications. The quality of non-randomized studies was assessed by the modified version of the Newcastle-Ottawa Scale (NOS; Stang, 2010). This scale assesses the quality of the study through five dimensions: sample representativeness, sample size, comparability between respondents and non-respondents, ascertainment of insomnia, stress, anxiety, and depression, and statistical quality (Supplementary Table 1). Studies were judged to be at low risk of bias (≥3 points) or high risk of bias (<3 points). The third reviewer (N.Z.) adjudicates all discrepancies.

### Data analysis

Prevalence estimates of insomnia, stress, anxiety, and depression were calculated by pooling the study-specific estimates using random-effects meta-analysis. It accounted for between study heterogeneity. The 95% confidence intervals of the studies were calculated by the Clopper Pearson method, which considered the asymmetry. Study heterogeneity was assessed by standard χ^2^-tests and *I*^2^ statistics. Heterogeneity values ≥75% indicated considerable heterogeneity. The characteristics of different study levels were grouped, and hierarchical meta-analysis and meta-regression were carried out (van Houwelingen et al., 2002). The impact of individual studies on the estimation of overall prevalence was explored by sensitivity analysis. Bias secondary to study effects was studied by funnel diagram and Egger's test. All analysis were performed using R Foundation for Statistical Computing (version 4.1.1; Computing, 2022). The statistical test of all studies was two-sided and used a significance threshold of *P* < 0.05. This study is registered on PROSPERO, number CRD42021253821.

### Role of the funding source

The funder of the study had no role in study design, data collection, data analysis, data interpretation, or writing of the report.

### Patient and public involvement

The development of the research question was informed by the mental health status among frontline HCWs during the COVID-19 pandemic. Patients were not advisers in this study, nor were they involved in the design, recruitment or conduct of the study. Results of this study will be made publicly available through open-access publication where study participants may access them.

## Results

### Study characteristics

**Figure 1:**
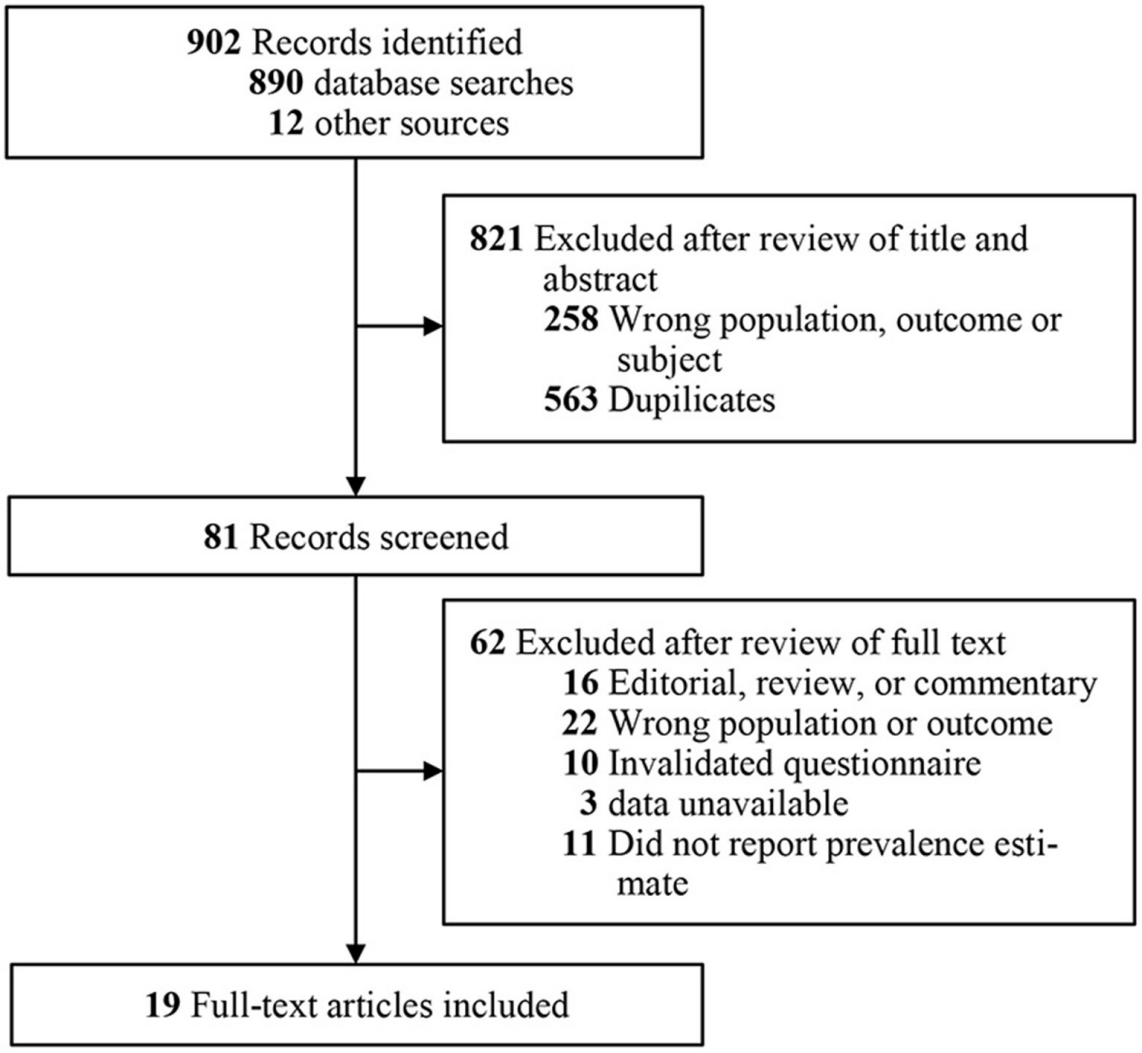
Flow diagram for identifying studies and selection.

Seventeen cross-sectional studies (*n* = 11,727), one cohort study (*n* = 4,804), and one RCT study (*n* = 482) involving 17,013 individuals were included in this study (Figure 1). The study involved 12 countries, including one in North America, one in South America, five in Europe, four in Asia, and one in Africa. Sixteen studies recruited participants from first frontline physicians and nurses, while three recruited participants exclusively from physicians. There were an average of 895 participants per study, ranging from 98 to 4,804. Thirteen studies assessed insomnia using the Insomnia Severity Index (ISI), 2 used the Pittsburgh Sleep Quality Index (PSQI), and one used the Sleep Condition Indicator (SCI). Five studies assessed stress using the Depression, Anxiety and Stress Scale (DASS-21), two used the 10-item Perceived Stress Scale (PSS-10), one used the 22-item Impact of Event Scale–Revised (IES-R), one used the Effort Reward Imbalance (ERI), and one used the 4-item Primary Care PTSD screen (PC-PTSD). Nine studies assessed anxiety using the Generalized Anxiety Disorder 7-item (GAD-7), five used the Depression, Anxiety and Stress Scale (DASS-21), two used the Goldberg depression and anxiety scale (GADS), one used the Effort Reward Imbalance (ERI). Eight studies assessed for depression using the Patient Health Questionnaire-9 (PHQ-9), five used the Depression, Anxiety and Stress Scale (DASS-21), two used the Goldberg Depression and Anxiety Scale (GADS), two used the 2-item Patient Health Questionnaire (PHQ-2), one used the Hospital Anxiety and Depression Scale (HADS), and 1 used the Patient Health Questionnaire-8 (PHQ-8). All studies were evaluated by the NOS quality assessment criteria, two studies received five points, six received four points, five received three points, and six received two points (Table 2, Supplementary Table 2).

**Table 2:** Selected characteristics of the 19 studies included in this systematic review and meta-analysis.

**References**	**Country**	**Survey period**	**Specialty**	**Number of participants**	**Age, *y***	**Men, No. (%)**	**Study design (survey methods)**	**Diagnostic methods**	**Outcome definition**	**NOS**
Giardino et al. (2020)	Argentina	June 5, 2020 to June 25, 2020	Physicians, physicians in trainee, psychologists	1,059	Mean (SD), 41.7 (10.7)	287 (27.1)	Multicenter cross-sectional study (online survey)	ISI GADS	≥8 A ≥ 5 D ≥ 2	2
Wang L. Q. et al. (2020)	China	February 26, 2020 to March 3, 2020	Physicians, nurses	274	NR	62 (22.6)	Cross-sectional study (online survey)	PSQI PHQ-9 GAD-7	≥6 ≥5 ≥5	4
Alshekaili et al. (2020)	Oman	April 8, 2020 to April 17,2020	Physicians, nurses, allied health	574	Mean (SD), 36.3 (6.5)	228 (20.0)	Multicenter cross-sectional online study (online survey)	ISI DASS	≥8 S ≥ 16 A ≥ 8 D ≥ 10	3
Elkholy et al. (2021)	Egypt	April 2020 to May 2020	Physicians, nurses, non-specialized nurse	502	No. (%) ≤ 30 y: 232 (46.2)	251 (50.0)	Multicenter cross-sectional study (online survey)	ISI PHQ-9 GAD-7 PSS-10	≥8 ≥5 ≥5 ≥9	4
Lee et al. (2021)	Korean	June 22 2020 to July 8, 2020	Physicians, nurses	406	No. (%) < 40 y: 225 (55.4)	115 (28.3)	Cross-sectional study (online survey)	ISI PHQ-9 GAD-7	≥8 ≥10 ≥5	4
Haravuori et al. (2020)	Finland	June 4, 2020 to June 26, 2020	Physicians, nurses, psychologists	4,804	Mean (SD), 44.2 (11.3)	538 (11.4)	Prospective multicenter cohort study (online survey)	ISI PHQ-2 OASIS	≥8 ≥3 ≥8	2
Liu et al. (2020)	China	March 7, 2020 to March 17, 2020	Obstetrician, midwive	2,126	NR	49 (2.3)	Multicenter cross-sectional study (online survey)	ISI PHQ-9 GAD-7	≥8 ≥5 ≥5	2
Almater et al. (2020)	Saudi Arabia	28 March 2020 to 4 April 2020	Ophthalmologists	107	Mean (SD), 32.9 (9.6)	60 (56.1)	Cross-sectional study (online survey)	ISI PHQ-9 GAD-7 PSS-10	≥8 ≥5 ≥5 ≥9	2
Cui et al. (2020)	China	February 1, 2020 to February 19, 2020	Female nurses	334	No. (%) ≤ 30 y: 202 (54.0)	NR	Cross-sectional online study (online survey)	ISI PHQ-9 GAD-7	≥8 ≥5 ≥5	3
Lai et al. (2020)	China	January 29, 2020 to February 3, 2020	Physicians, nurses	1,257	No. (%) ≤ 30 y: 605 (48.1)	293 (23.3)	Multicenter cross-sectional study (online survey)	ISI PHQ-9 GAD-7 IES-R	≥8 ≥5 ≥5 ≥9	5
Magnavita et al. (2020a)	Italy	April 27, 2020 to May 27, 2020	Anesthetists	155	No. (%) ≤ 35 y: 118 (76.7)	74 (47.8)	Cross-sectional study (online survey)	SCI ERI GADS	≥16 ≥2 A ≥ 5 D ≥ 2	2
Shechter et al. (2020)	United States	April 9, 2020 to Apr 24, 2020	Physicians, residents/fellows, nurses	657	No. (%) ≤ 35 y: 347 (76.7)	143 (19.9)	Multicenter cross-sectional study (online survey)	PHQ-2 GAD-2 PC-PTSD	≥3 ≥3 ≥3	3
Tiete et al. (2020)	Belgium	April 17 2020 to May 25, 2020	Physicians, nurses	647	No. (%) ≤ 30 y: 149 (23.0)	140 (21.6)	Multicenter cross-sectional study (online survey)	ISI DASS-21	≥8 S ≥ 16 A ≥ 8 D ≥ 10	3
Youssef et al. (2020)	Egypt	April 1, 2020 to April 15, 2020	Physicians, nurses	540	Mean (SD), 37.3 (9.2)	294 (94.4)	Multicenter cross-sectional study (online survey)	ISI DASS-21	≥8 S ≥ 16 A ≥ 8 D ≥ 10	5
Azoulay et al. (2021)	France	October 30, 2020 to December 1, 2020	Physicians, nurses	845	NR	274 (32.4)	Multicenter cross-sectional study (online survey)	HADS	A ≥ 8 D ≥ 8	4
Di Mattei et al. (2021)	Italy	May 9, 2020 to July 13, 2020	Physicians, nurses, psychologists, healthcare assistants	1,055	Mean (SD), 44.7 (11.3)	256 (24.3)	Multicenter cross-sectional study (online survey)	ISI DASS-21	≥8 S ≥ 16 A ≥ 8 D ≥ 10	3
Fiol-DeRoque et al. (2021)	Spain	May 14, 2020 to July 25, 2020	Physicians, nurses, nurse assistants	482	Mean (SD), 41.3 (10.4)	81 (16.8)	RCT (Online and mobile phone survey)	ISI DASS-21	≥8 S ≥ 16 A ≥ 8 D ≥ 10	4
Guo et al. (2021)	China	May 15, 2020 to May 31, 2020	Physicians, nurses	1,091	No. (%) ≤ 45 y: 888 (81.4)	356 (32.6)	Multicenter cross-sectional study (online survey)	PSQI PHQ-9 GAD-7	≥6 ≥5 ≥5	4
Wright et al. (2021)	United States	April 1, 2020 to May 7, 2020	Physicians, nurses	98	Mean (SD), 42.9 (11.0)	NR	Cross-sectional study (online survey)	PHQ-8 GAD-7	≥10 ≥5	2

A, Anxiety; D, Depression; DASS-21, The Depression, Anxiety and Stress Scale; ERI, Effort Reward Imbalance; GAD-7, Generalized Anxiety Disorder 7-item; GAD-2, 2-item Generalized Anxiety Disorder Scale; GADS, Goldberg depression and anxiety scale; HADS, Hospital Anxiety and Depression Scale; S, Stress; ISI, The seven-item Insomnia Severity Index; IES-R, 22-item Impact of Event Scale–Revised; NOS, Newcastle-Ottawa Score; OASIS, Overall Anxiety Severity and Impairment Scale; PC-PTSD, 4-item Primary Care PTSD screen; PHQ-9, Patient Health Questionnaire-9; PHQ-2, 2-item Patient Health Questionnaire; PSS-10, 10-item Perceived Stress Scale; PSQI, Pittsburgh Sleep Quality Index; NR, not reported; S, Stress; SCI, Sleep Condition Indicator.

### Prevalence of insomnia or insomnia symptoms

A meta-analysis of insomnia or insomnia symptoms among frontline HCWs during the COVID-19 pandemic reported by 16 studies showed a summary prevalence of 42.9% (7,068/15 413 individuals, 95% CI, 33.9–51.9%). There was significant evidence of between study heterogeneity (*Q* = 2,106.2, *P* < 0.001, τ^2^ = 0.03, *I*^2^ = 99.0%). A sensitivity analysis showed that no individual study had an impact on the overall prevalence estimate of more than 3% (Supplementary Table 3). The estimated values were stratified by screening instrument and outcome definition. Summary prevalence estimates ranged from 43.5% of the ISI (6,121/13,893 individuals, 95% CI, 35.4–51.6%, *Q* = 1,149.3, τ^2^ = 0.02, *I*^2^ = 99.0%) and 49.3% of the PSQI (914/1,365 individuals, 95% CI, −8.6–107.2%, *Q* = 185.1, τ^2^ = 0.17, *I*^2^ = 100.0%; Table 3).

**Table 3:**
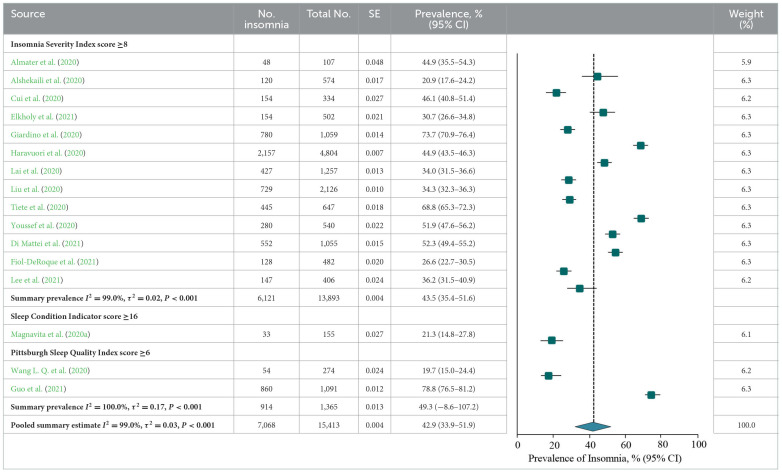
Meta-analysis of the prevalence of insomnia or insomnia symptoms among frontline HCWs during the COVID-19 pandemic.

Studies are stratified by screening modality. The vertical dashed lines indicate the pooled summary estimate (95% CI) for all studies and horizontal lines indicate 95% confidence intervals of the estimate.

According to study-level characteristics, there were statistically significant differences in prevalence estimates between multi-center studies [6,504/13,655 (49.0%; 95% CI, 37.7–60.4%)] and non-multi-center studies [564/1,758 (32.1%; 95% CI, 23.6–40.7%)] (*Q* = 0.02, *P* = 0.02). Studies were further stratified by continent or region. Studies performed in the South America [780/1,059 (73.7%; 95% CI, 70.9–76.4%)] has the highest prevalence, which is significantly different from those performed on other continents (*Q* = 47.1, *P* < 0.001). When studies were stratified by study period and individual specialty, prevalence estimates were not statistically significant differences (*P* > 0.05; **Table 7**A). By NOS criteria, studies with more thorough descriptive statistics reporting [2,968/5,183; 54.2% (95% CI, 37.5–70.9%)] had higher prevalence estimates than those with less comprehensive descriptive statistics reporting [4,100/10,230; 34.2% (95% CI, 27.0–41.3%); *Q* = 4.7, *P* = 0.03]. The estimated prevalence was not significantly different when studies were stratified by sample representativeness, size, comparability between respondents and non-respondents, ascertainment of insomnia, or total NOS score (*P* > 0.05; **Table 8**A; details of the NOS appear in Supplementary Table 1).

### Prevalence of stress or stress symptoms

There were 10 studies on the prevalence of stress or stress symptoms among frontline HCWs during the COVID-19 pandemic. The meta-analysis pooling of the prevalence estimates was 53.0% (3,284/5,976 individuals, 95% CI, 41.1–64.9%), with significant evidence of between study heterogeneity (*Q* = 897.7, *P* < 0.001, τ^2^ = 0.04, *I*^2^ = 99.0%). A sensitivity analysis showed that the impact of individual studies on the estimation of the overall prevalence estimate was <6% (Supplementary Table 4). The estimated values were stratified according to screening instrument and outcome definition, showing that summary prevalence estimates ranged from 78.3% for the PSS-10 (6,121/13,893 individuals, 95% CI, 67.5–89.0%, *Q* = 5.8, τ^2^ = 0.01, *I*^2^ = 83.0%) and 41.1% for the DASS-21 (1,453/3,298 individuals, 95% CI, 28.6–53.7%, *Q* = 234.6, τ^2^ = 0.02, *I*^2^ = 98.0%; Table 4).

**Table 4:**
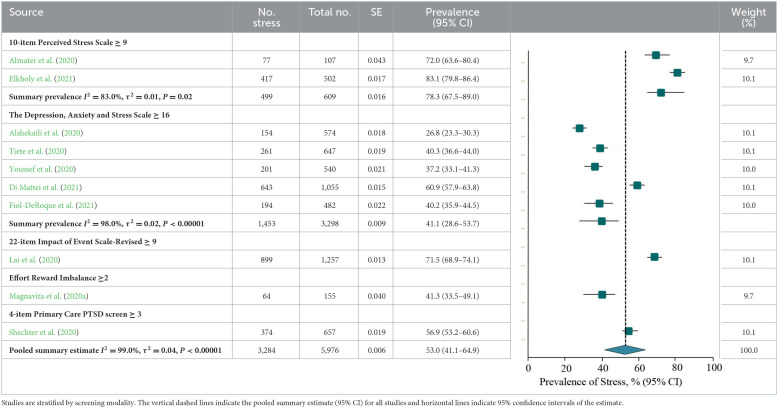
Meta-analysis of the prevalence of stress or stress symptoms among frontline HCWs during the COVID-19 pandemic.

Studies are stratified by screening modality. The vertical dashed lines indicate the pooled summary estimate (95% CI) for all studies and horizontal lines indicate 95% confidence intervals of the estimate.

According to the study-level characteristics of the study period, study design, individuals‘ specialty, and continent or region, prevalence estimates were not significantly different (*P* > 0.05; **Table 7**B). Prevalence estimates of studies with more sample representative reporting [3,207/5,869; 50.9% (95% CI, 38.3–63.6%)] were lower than those with less sample representative reporting [77/107; 72.0% (95% CI, 63.6–80.4%); *Q* = 7.4, *P* = 0.007] when evaluated by NOS criteria. There was a significant difference (*Q* = 4.3, *P* = 0.04) between the prevalence estimates of studies with lower valid of ascertainment [261/647 (40.3%; 95% CI, 36.6–44.0%)] and more valid of ascertainment [3,023/5,329 (54.4%; 95% CI, 41.6–67.2%)]. The prevalence estimates were not significantly different when studies were stratified by comparability between respondent and non-respondent, sample size, descriptive statistics reporting, or total NOS score (*P* > 0.05; **Table 8**; details of the NOS appear in Supplementary Table 1).

### Prevalence of anxiety or anxiety symptoms

The meta-analysis of anxiety or anxiety symptoms among frontline HCWs during the COVID-19 pandemic reported by the 19 studies showed a summary prevalence of 43.0% (6,509/17,013 individuals, 95% CI, 33.8–52.3%), and there was significant heterogeneity between studies (*Q* = 3,019.0, *P* < 0.001, τ^2^ = 0.04, *I*^2^ = 99.0%). A sensitivity analysis showed that the impact of individual studies on the overall prevalence estimate was <7% (Supplementary Table 5). According to stratification by instrument and outcome definition, summary prevalence estimates ranged from 44.0% for the GAD-7 (2,644/6,195 individuals, 95% CI, 31.6–56.5%, *Q* = 818.0, τ^2^ = 0.04, *I*^2^ = 99.0%), 43.2% for the DASS-21 (1,373/3,298 individuals, 95% CI, 34.1–51.5%, *Q* =98.3, τ^2^ = 0.01, *I*^2^ = 96.0%), and 46.4% for the GADS (835/1,214 individuals, 95% CI, −12.8–105.6%, *Q* = 341.3, τ^2^ = 0.18, *I*^2^ = 100.0%; Table 5).

**Table 5:**
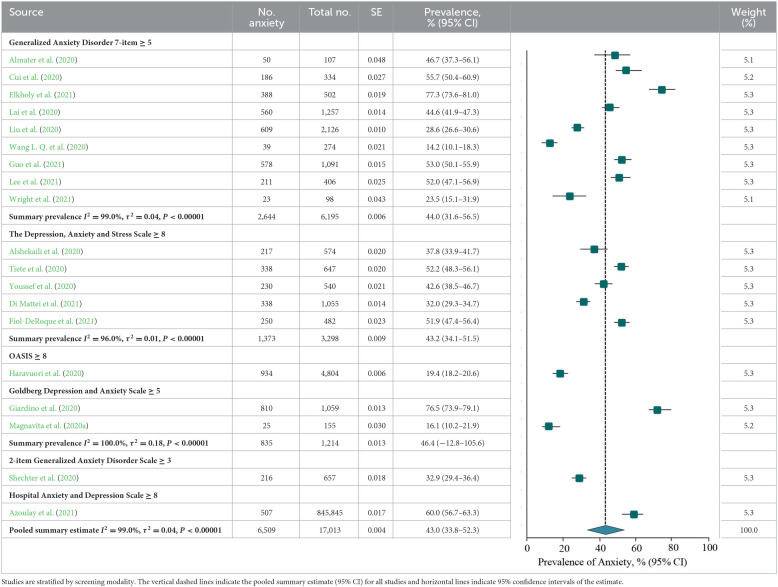
Meta-analysis of the prevalence of anxiety or anxiety symptoms among frontline HCWs during the COVID-19 pandemic.

Studies are stratified by screening modality. The vertical dashed lines indicate the pooled summary estimate (95% CI) for all studies and horizontal lines indicate 95% confidence intervals of the estimate.

According to study-level characteristics, the prevalence of studies performed in South America [810/1,059 (76.5%; 95% CI, 73.9–79.1%)] was higher than that on other continents, and the difference was statistically significant (*Q* = 152.9, *P* < 0.001). When studies were stratified by study design, period, and individuals specialty, the prevalence estimates of anxiety were not significantly different (*P* > 0.05; **Table 7**C). There were no statistically significant differences in prevalence estimates of six-dimensional stratification when evaluated by NOS criteria (*P* > 0.05; **Table 8**C; details of the NOS appear in Supplementary Table 1).

### Prevalence of depression or depressive symptoms

A meta-analysis of depression or depressive symptoms among frontline HCWs during the COVID-19 pandemic reported by the 19 studies yielded a summary prevalence of 44.6% (7,452/17,013 individuals, 95% CI, 36.1–53.1%), with significant difference between study heterogeneity (*Q* = 2,410.8, *P* < 0.001, τ^2^ = 0.04, *I*^2^ = 99.0%). Sensitivity analysis suggested that no individual study had an impact on the overall prevalence estimate of more than 5% (Supplementary Table 6). According to stratification by instrument and outcome definition, prevalence estimates ranged from 46.3% for the PHQ-9 (2,896/6,097 individuals, 95% CI, 32.0–60.5%, *Q* = 975.6, τ^2^ = 0.04, *I*^2^ = 99.0%), 45.8% for the DASS-21 (1,485/3,298 individuals, 95% CI, 37.0–54.5%, *Q* =105.5, τ^2^ = 0.01, *I*^2^ = 96.0%), 55.5% for the GADS (904/1,214 individuals, 95% CI, 5.2–105.7%, *Q* = 173.9, τ^2^ = 0.13, *I*^2^ = 99.0%), and 39.8% for the PHQ-2 (1,849/5,461 individuals, 95% CI, 24.1–55.5%, *Q* = 62.4, τ^2^ = 0.01, *I*^2^ = 98.0%; Table 6).

**Table 6:**
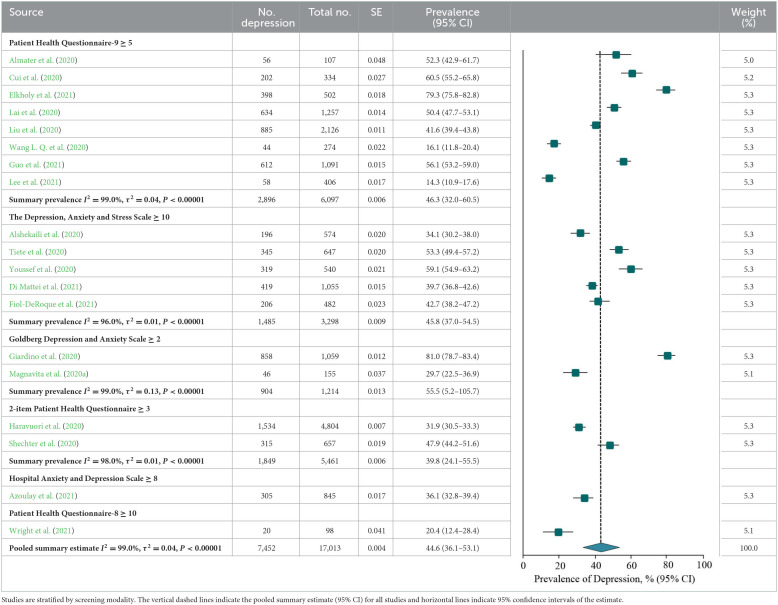
Meta-analysis of the prevalence of depression or depressive symptoms among frontline HCWs during the COVID-19 pandemic.

Studies are stratified by screening modality. The vertical dashed lines indicate the pooled summary estimate (95% CI) for all studies and horizontal lines indicate 95% confidence intervals of the estimate.

The estimated values were stratified according to study-level characteristics. The prevalence estimates between multi-center studies [5,286/10,353 (52.6%; 95% CI, 42.7–62.6%)] and non-multi-center studies [2,141/6,660 (33.3%; 95% CI, 23.7–42.9%); *Q* = 7.5, *P* = 0.006] were statistically significance. Studies were further stratified by continent or region. Studies performed in the South America [858/1,059 (81.0%; 95% CI, 78.7–83.4%)] had the highest prevalence, which was significantly different from those performed on other continents (*Q* = 187.8, *P* < 0.001). The prevalence estimates were not significantly different when studies were stratified by study period and individual specialty (*P* > 0.05; Table 7D). When assessed by NOS criteria, studies with more thorough descriptive statistics reporting [3,005/5,183; 56.5% (95% CI, 45.3–67.7%)] had higher prevalence than those with less thorough descriptive statistics reporting [4,422/11,830; 37.7% (95% CI, 28.9–46.5%); *Q* = 6.7, *P* = 0.01]. There were no statistically significant differences in prevalence estimates between studies stratified by sample representativeness, sample size, comparability between respondents and non-respondents, ascertainment of depression, or total NOS score (*P* > 0.05; Table 8; details of the NOS appear in Supplementary Table 1).

**Table 7:**
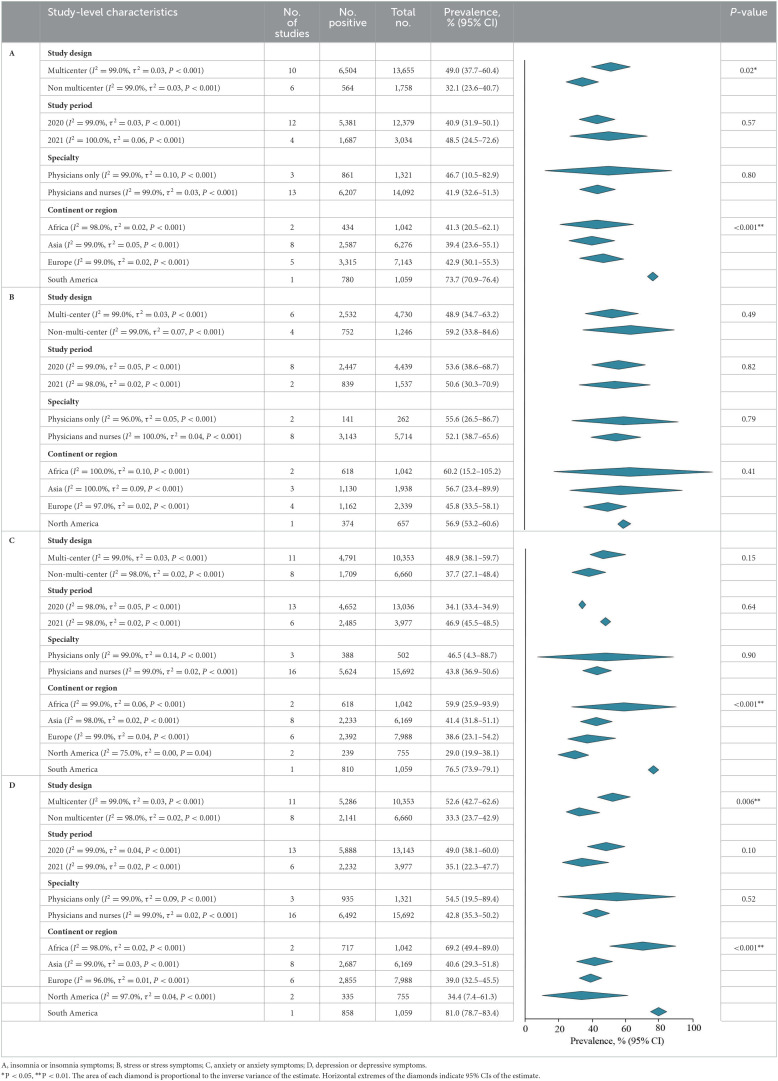
Meta-analysis of the prevalence of insomnia, stress, anxiety, and depression among frontline HCWs during the COVID-19 pandemic stratified by study-level characteristics.

A, insomnia or insomnia symptoms; B, stress or stress symptoms; C, anxiety or anxiety symptoms; D, depression or depressive symptoms.

^*^P < 0.05, ^**^P < 0.01. The area of each diamond is proportional to the inverse variance of the estimate. Horizontal extremes of the diamonds indicate 95% CIs of the estimate.

**Table 8:**
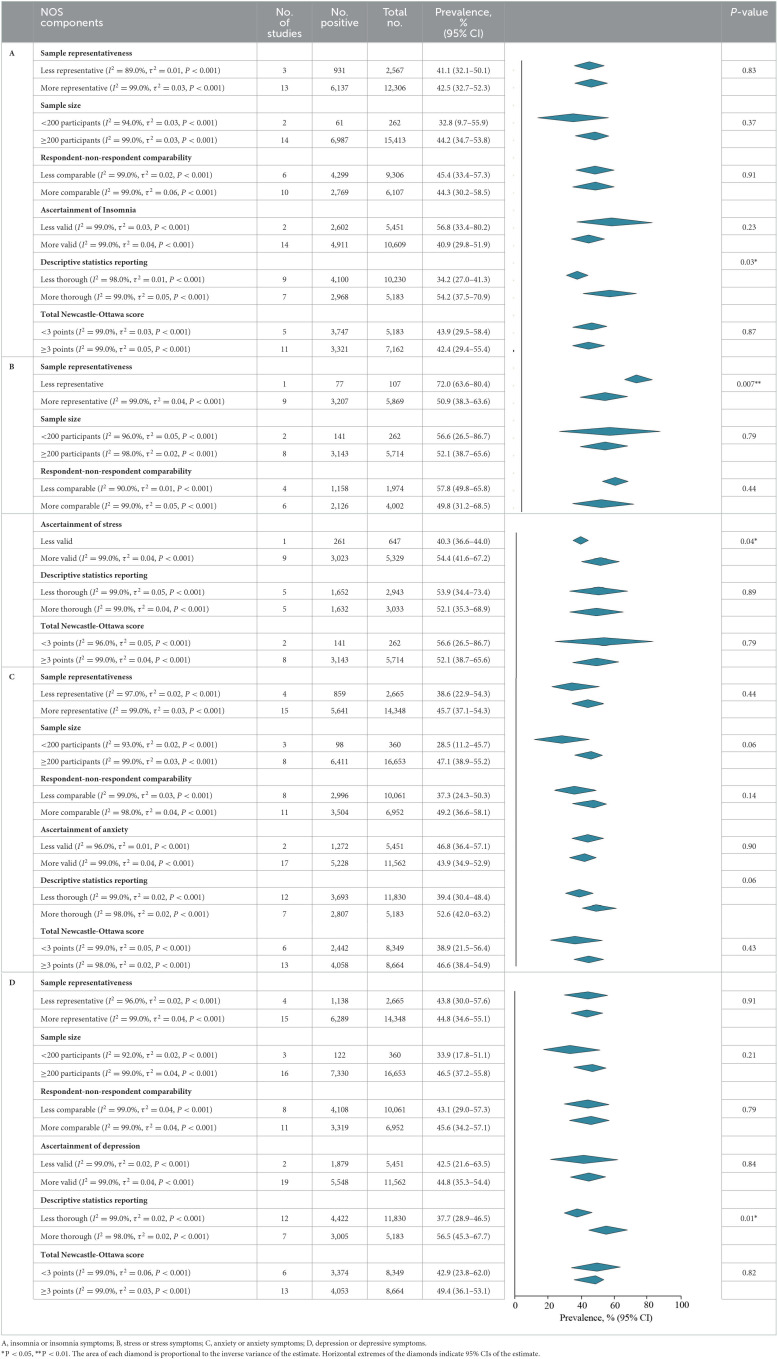
Meta-analysis of the prevalence of insomnia, stress, anxiety, and depression among frontline HCWs during the COVID-19 pandemic stratified by NOS components and total score.

A, insomnia or insomnia symptoms; B, stress or stress symptoms; C, anxiety or anxiety symptoms; D, depression or depressive symptoms.

^*^P < 0.05, ^**^P < 0.01. The area of each diamond is proportional to the inverse variance of the estimate. Horizontal extremes of the diamonds indicate 95% CIs of the estimate.

### Assessment of publication bias

Visual examination of the funnel plot reporting on insomnia, stress, anxiety, and depression revealed minimal asymmetry (Supplementary Figures 1–4), suggesting that there was no significant publication bias (*P* = 0.75; *P* = 0.69; *P* = 0.16; *P* = 0.51 using the Egger test).

### Discussion

The systematic review and meta-analysis of 19 studies involving 17,013 frontline HCWs in 12 countries during the COVID-19 pandemic demonstrated that 42.9% (range, 33.8–52.3%) and 53.0% (range, 41.1–64.9%) of workers reported insomnia and stress, and 43.0% (range, 33.8–52.3%) and 44.6% (range, 36.1–53.1%) screened positive for anxiety and depression, respectively. This data is significantly higher than the prevalence of anxiety (31.9%), depression (33.7%), and stress (29.6%) among general population population during the COVID-19 pandemic (Salari et al., 2020), and is similar to the prevalence of depression (45.0%), anxiety (47%), and insomnia (34%) among patients with COVID-19 (Deng et al., 2021). Compared with the small pandemic of SARS in 2002 and MARS in 2012, COVID-19 has a greater psychological impact on HCWs (Boden et al., 2021; Magnavita et al., 2021). This is closely related to the unknown characteristics of COVID-19, the high risk of exposure to infection, and multiple work pressures. However, Magnavita et al. (2020b) pointed out that the frequency of anxiety and depression among HCWs during the early stage of the COVID-19 pandemic was not higher than the commonly recorded during periodic checks in the years preceding the epidemic, which may be related to the severity of the epidemic in the region or the type of work involving front-line or non-front-line workers. These findings are concerning given that the development of mental health among HCWs has been linked to an increased long-term risk of future mental health diseases.

It is important to note that all participants were assessed through online self-report inventories rather than the gold-standard diagnostic clinical interviews. The sensitivity and specificity of these instruments for estimating symptoms of insomnia, stress, anxiety, and depression vary substantially (Supplementary Table 7). In the evaluation of insomnia and stress, the sensitivity of the ISI (Morin et al., 2011; 99%, 95% CI, 97–100%) and IES-R (Lee, 2012; 86%, 95% CI, 67–94%) was higher than that of the others. However, that of ESS (Siegrist et al., 2014; 76%, 95% CI, 63–86%, 20%) and PC-PTSD (Li et al., 2019; 57%, 95% CI, 45–68%) are the least. When estimating anxiety symptoms, the specificity of the HADS-A (Hitchon et al., 2020) was 78% (95% CI, 69–85%), which was lower than that of the others. In contrast, the PHQ-9 (Williams et al., 2002) has high sensitivity (88%, 95% CI, 74–96%) and specificity (88%, 95% CI, 85–90%) for diagnosing major depression and has been proven to be comparable with the administered assessments by clinicians, whereas the GHQ (Williams et al., 2002) has low specificity (66%, 95% CI, 57–74%). In addition, all self-report measures were conducted online because of the COVID-19 pandemic which may affect the accurate evaluation of symptoms.

The sub-analysis revealed potentially important differences, such as study period, specialty and region. The prevalence of stress and depression is higher in 2020 and that of insomnia and anxiety is higher in 2021. This probably reflects the already established psychological problems of the population that have changed with the evolution of COVID-19 (Simon et al., 2020; Kola et al., 2021). HCWs changed from stress and depression during the outbreak of the COVID-19 pandemic to mild psychological problems of sleep and anxiety with the normalization of COVID-19 prevention and control. In the HCWs specialty, the prevalence of psychological problems of physicians is higher than that of other HCWs. This is related to the working hours and strength of the physicians at the frontline of COVID-19 (Elbay et al., 2020). By region, the prevalence of HCWs in South America is higher, while the prevalence of anxiety and depression in North America is lower, which is related to local economic level, contact tracing, isolation and management, and other measures (Fitzpatrick et al., 2020; Goularte et al., 2021).

Furthermore, according to NOS criteria analysis, variation in sample size contributed significantly to the observed heterogeneity in the study. Studies with fewer participants usually produced more extreme prevalence estimates of stress, indicating publication bias. However, for insomnia, anxiety, and depression, studies with more participants usually produced more extreme prevalence estimates. These differences were partly captured by the NOS score, which assessed the risk of bias in each study. Studies with a higher risk of bias produced higher prevalence estimates of insomnia and stress, while studies with a lower risk of bias produced higher prevalence estimates of anxiety and depression. These findings may be related to the heterogeneity between studies on study design (i.e., multicenter vs. non multicenter), online surveys, study period of COVID-19, positions of frontline HCWs, and severity of regional pandemic.

Most opinions show that psychological strategies are the mediating factor to change these outstanding problems, although the stressors (COVID-19 pandemic) cannot be changed (Mediavilla et al., 2022). Importantly, increasing the so-called social support barriers (i.e., factors that increase the use of social support, even when available) might also contribute to improving the mental health of HCWs (Thoresen et al., 2014). With mounting evidence suggesting an association between reported discrimination against COVID-19 and poor mental health outcomes among HCWs, mental health strategies at the community level could take the form of anti-stigma campaigns (Taylor et al., 2020). At the policy level, addressing various common problems reported by HCWs, including increased workload, shortage of protective equipment, or lack of standardized operating procedures, may also enable HCWs to help reduce the negative consequences of mental health problems (Erquicia et al., 2020).

## Limitations

When interpreting the results of this study, several limitations should be considered. First, a substantial amount of the heterogeneity among frontline HCWs remained unexplained by the variables examined. We attempted to reduce these impacts by assessing and reporting the risk of bias. Second, most studies were observational and lacked enough cohort studies; thus, they were vulnerable to the effects of confounding factors. It is necessary to explore the psychological impacts on frontline HCWs during the COVID-19 pandemic over a longer and more prospective period (Shanafelt et al., 2020; Pan et al., 2021). It can be helpful in clarifying the mental state of this population in the future. Third, fewer studies can be included in this meta-analysis because of the special background of the COVID-19 pandemic, which is difficult to analyze through different dimensions of mental health problems. At present, most studies focus on how to control the pandemic, but the mental health of frontline HCWs is easy to be ignore (Greenberg et al., 2020; The, 2020; de Vroege and van den Broek, 2021).

## Conclusions

This systematic review and meta-analysis showed that the COVID-19 pandemic have significant effects on mental health among frontline HCWs. The overall prevalence of insomnia, stress, anxiety and depression among frontline HCWs is high. Therefore, the health policy-makers should pay attention to and respond to the mental health problems of frontline HCWs in the context of public health emergencies.

## Data availability statement

The original contributions presented in the study are included in the article/Supplementary material, further inquiries can be directed to the corresponding authors.

## Author contributions

JT, XS, JZ, and CH conceived the study aims. JT and WL undertook screening for the review. JZ and WY extracted data and checked it. YP and NZ undertook risk of bias assessment. JT, XS, and JZ developed the analysis plan. CH oversaw data analysis. JT drafted the initial manuscript. JZ edited the initial draft. XS verified the data. JT, XS, and CH obtained funding for the study. All authors conceptualized and approved the research protocol, which outlined the aims and study methods used here, provided critical comments on the manuscript, had access to the data, and approved the final version of the manuscript.
