# Supplementary material for: Effects of COVID-19 pandemic on mental health among frontline healthcare workers: A systematic review and meta-analysis
Source: Front Psychol. 2023 Jan 27;13:1096857. doi: 10.3389/fpsyg.2022.1096857 (PMC9912473; doi:10.3389/fpsyg.2022.1096857)
Supplement: Supplementary file 1 [file Data_Sheet_1.PDF]

Supplemental Material Content

eTable 1. Modified Newcastle-Ottawa risk of bias scoring guide.

eTable 2. Newcastle-Ottawa Risk of Bias Assessment.

eTable 3. Sensitivity Analysis of the Prevalence of Insomnia or Insomnia Symptoms Among Frontline Healthcare Workers During the COVID-19 Pandemic.

eTable 4. Sensitivity Analysis of the Prevalence of Stress or Stress Symptoms Among Front-line Healthcare Workers During the COVID-19 Pandemic.

eTable 5. Sensitivity Analysis of the Prevalence of Anxiety or Anxiety Symptoms Among Frontline Healthcare Workers During the COVID-19 Pandemic.

eTable 6. Sensitivity Analysis of the Prevalence of Depression or Depressive Symptoms Among Frontline Healthcare Workers During the COVID-19 Pandemic.

eTable 7. Sensitivities and Specificities of Commonly Used Instruments for Diagnosing In-somni, Stress, Anxiety, and Depression.

eFigure 1. Assessment of Small Study Effects by Funnel Plot for the 16 Studies Reporting on Insomnia or Insomnia Symptoms Prevalence.

eFigure 2. Assessment of Small Study Effects by Funnel Plot for the 10 Studies Reporting on Stress or Stress Symptoms Prevalence.

eFigure 3. Assessment of Small Study Effects by Funnel Plot for the 19 Studies Reporting on Anxiety or Anxiety Symptoms Prevalence.

eFigure 4. Assessment of Small Study Effects by Funnel Plot for the 19 Studies Reporting on Depression or Depressive Symptoms Prevalence.

eTable 1. Modified Newcastle-Ottawa risk of bias scoring guide.

Sample representativeness	
1 point	Population contained multiple hospitals in multiple regions.
0 points	Population contained either a single hospital, a single regions, or both.
Sample size	
1 point	Sample size was greater than or equal to 200 participants.
0 points	Sample size was less than 200 participants.
Non-respondents	
1 point	Comparability between respondent and non-respondent characteristics was established with a satisfactory response rate.
0 points	The comparability between respondents and non-respondents was unsatisfactory, the response rate was unsatisfactory, or there was no description of the response rate or the characteristics of the responders or non-responders.
Ascertainment of insomnia, stress, anxiety, and depression	
1 point	The study employed a commonly used measurement tool with a valid cutoff score.
0 points	The study employed an infrequently used measurement tool, a commonly used measurement tool with an invalid cutoff score, or any tool with published sensitivity/specificity values <70%.
Quality of descriptive statistics reporting	
1 point	The study reported descriptive statistics to describe the population (e.g., age, sex, class year) with proper measures of dispersion.
0 points	The study did not report descriptive statistics, incompletely reported descriptive statistics, or did not report measures of dispersion.

eTable 2. Newcastle-Ottawa Risk of Bias Assessment.

First Author, Year	Representativeness	size	Comparability	Outcome	Statistics	Total
Almater et al, [1] 2020	0	0	0	1	1	2
Alshekaili et al, [2] 2020	1	0	1	1	0	3
Cui et al, [3] 2020	0	1	1	1	0	3
Elkholy et al, [4] 2020	1	1	1	1	0	4
Giardino et al, [5] 2020	1	1	0	0	0	2
Haravuori et al, [6] 2020	1	1	0	0	0	2
Lai et al, [7] 2020	1	1	1	1	1	5
Liu et al, [8] 2020	0	1	0	1	0	2
Magnavita et al, [9] 2020	1	0	0	1	0	2
Shechter et al, [10] 2020	1	1	0	1	0	3
Tiete et al, [11] 2020	1	0	1	1	0	3
Wang et al, [12] 2020	1	1	1	1	0	4
Youssef et al, [13] 2020	1	1	1	1	1	5
Azoulay et al, [14] 2021	1	1	1	1	0	4
Di Mattei et al, [15] 2021	1	1	0	1	0	3
Fiol-DeRoque et al, [16] 2021	1	0	1	1	1	4
Guo et al, [17] 2021	1	0	1	1	1	4
Lee et al, [18] 2021	1	0	1	1	0	4
Wright et al, [19] 2021	0	1	0	1	0	2

eTable 3. Sensitivity Analysis of the Prevalence of Insomnia or Insomnia Symptoms Among Frontline Healthcare Workers During the COVID-19 Pandemic.

First Author, Year	Prevalence (%)	95% CI		Tau^2	I^2
		Lower	Upper		
Omitted Almater et al, [1] 2020	45.9	45.1	46.7	0.68	99%
Omitted Alshekaili et al, [2] 2020	46.8	46.0	47.6	0.60	99%
Omitted Cui et al, [3] 2020	45.9	45.1	46.7	0.68	99%
Omitted Elkholy et al, [4] 2020	46.4	45.6	47.2	0.66	99%
Omitted Giardino et al, [5] 2020	43.8	43.0	44.6	0.55	99%
Omitted Haravuori et al, [6] 2020	46.3	45.3	47.2	0.68	99%
Omitted Lai et al, [7] 2020	46.9	46.1	47.7	0.67	99%
Omitted Liu et al, [8] 2020	47.7	46.9	48.6	0.67	99%
Omitted Magnavita et al, [9] 2020	46.1	45.3	47.1	0.61	99%
Omitted Tiete et al, [11] 2020	44.9	44.1	45.7	0.59	99%
Omitted Wang et al, [12] 2020	46.3	45.5	47.1	59.4	99%
Omitted Youssef et al, [13] 2020	45.6	44.8	46.4	0.67	99%
Omitted Di Mattei et al, [15] 2021	45.4	44.6	46.2	0.67	99%
Omitted Fiol-DeRoque et al, [16] 2021	46.5	45.7	47.3	0.64	99%
Omitted Guo et al, [17] 2021	43.4	42.5	44.2	48.8	99%
Omitted Lee et al, [18] 2021	46.1	45.3	46.9	0.67	99%

eTable 4. Sensitivity Analysis of the Prevalence of Stress or Stress Symptoms Among Front-line Healthcare Workers During the COVID-19 Pandemic.

First Author, Year	Prevalence (%)	95% CI		Tau^2	I^2
		Lower	Upper		
Omitted Almater et al, [1] 2020	54.6	53.4	55.9	0.58	99%
Omitted Alshekaili et al, [2] 2020	57.9	56.6	59.3	0.49	98%
Omitted Elkholy et al, [4] 2020	52.4	51.1	53.7	0.40	98%
Omitted Lai et al, [7] 2020	50.5	49.1	52.0	0.58	98%
Omitted Magnavita et al, [9] 2020	55.3	54.0	56.6	0.63	99%
Omitted Shechter et al, [10] 2020	54.7	53.4	56.0	0.66	99%
Omitted Tiete et al, [11] 2020	56.7	55.4	58.1	0.62	99%
Omitted Youssef et al, [13] 2020	56.7	55.4	58.0	0.60	99%
Omitted Di Mattei et al, [15] 2021	53.7	52.3	55.1	0.65	99%
Omitted Fiol-DeRoque et al, [16] 2021	56.2	54.9	57.6	0.62	99%

eTable 5. Sensitivity Analysis of the Prevalence of Anxiety or Anxiety Symptoms Among Frontline Healthcare Workers During the COVID-19 Pandemic.

First Author, Year	Prevalence (%)	95% CI		Tau^2	I^2
		Lower	Upper		
Omitted Almater et al, [1] 2020	38.2	37.5	38.9	0.69	99%
Omitted Alshekaili et al, [2] 2020	38.2	37.5	39.0	0.69	99%
Omitted Cui et al, [3] 2020	37.9	37.1	38.6	0.67	99%
Omitted Elkholy et al, [4] 2020	37.0	36.3	37.8	0.55	99%
Omitted Giardino et al, [5] 2020	35.7	34.9	36.4	0.55	99%
Omitted Haravuori et al, [6] 2020	45.6	44.7	46.5	0.62	99%
Omitted Lai et al, [7] 2020	37.7	37.0	38.5	0.69	99%
Omitted Liu et al, [8] 2020	39.6	38.8	40.4	0.67	99%
Omitted Magnavita et al, [9] 2020	38.4	37.7	39.2	0.59	99%
Omitted Shechter et al, [10] 2020	38.4	37.7	39.2	0.68	99%
Omitted Tiete et al, [11] 2020	37.7	36.9	38.4	0.68	99%
Omitted Wang et al, [12] 2020	38.6	37.9	39.3	0.57	99%
Omitted Youssef et al, [13] 2020	38.1	37.3	38.8	0.69	99%
Omitted Azoulay et al, [14] 2021	37.1	36.3	37.8	0.66	99%
Omitted Di Mattei et al, [15] 2021	38.6	37.9	39.4	0.68	99%
Omitted Fiol-DeRoque et al, [16] 2021	37.8	37.1	38.6	0.68	99%
Omitted Guo et al, [17] 2021	37.2	36.5	38.0	0.68	99%
Omitted Lee et al, [18] 2021	37.8	37.1	38.6	0.68	99%
Omitted Wright et al, [19] 2021	38.3	37.6	39.0	0.65	99%

eTable 6. Sensitivity Analysis of the Prevalence of Depression or Depressive Symptoms Among Frontline Healthcare Workers During the COVID-19 Pandemic.

First Author, Year	Prevalence (%)	95% CI		Tau^2	I^2
		Lower	Upper		
Omitted Almater et al, [1] 2020	43.8	43.0	44.5	0.72	99%
Omitted Alshekaili et al, [2] 2020	44.1	43.4	44.9	0.72	99%
Omitted Cui et al, [3] 2020	43.5	42.7	44.2	0.70	99%
Omitted Elkholy et al, [4] 2020	42.7	42.0	43.5	0.58	99%
Omitted Giardino et al, [5] 2020	41.3	40.6	42.1	0.55	98%
Omitted Haravuori et al, [6] 2020	48.5	47.6	49.4	0.71	98%
Omitted Lai et al, [7] 2020	43.3	42.5	44.1	0.72	99%
Omitted Liu et al, [8] 2020	44.1	43.4	44.9	0.73	99%
Omitted Magnavita et al, [9] 2020	43.9	42.2	44.7	0.70	99%
Omitted Shechter et al, [10] 2020	43.6	42.9	44.4	0.73	99%
Omitted Tiete et al, [11] 2020	43.4	42.7	44.2	0.72	99%
Omitted Wang et al, [12] 2020	44.3	43.5	45.0	0.61	99%
Omitted Youssef et al, [13] 2020	43.3	42.6	44.1	0.70	99%
Omitted Azoulay et al, [14] 2021	44.2	43.4	45.0	0.72	99%
Omitted Di Mattei et al, [15] 2021	41.1	43.3	44.8	0.73	99%
Omitted Fiol-DeRoque et al, [16] 2021	43.8	43.1	44.6	0.73	99%
Omitted Guo et al, [17] 2021	43.0	42.2	43.7	0.71	99%
Omitted Lee et al, [18] 2021	44.5	43.8	45.3	0.58	99%
Omitted Wright et al, [19] 2021	43.9	43.2	44.7	0.66	99%

eTable 7. Sensitivities and Specificities of Commonly Used Instruments for Diagnosing Insomnia, Stress, Anxiety, and Depression.

Instrument	Cutoff	Sensitivity (%)	Specificity (%)
Insomnia			
ISI [20]	≥8	99 (97 to 100)	92 (89 to 95)
PSQI [21]	≥6	100 (83 to 100)	49 (36 to 63)
ESS [21]	≥10	76 (63 to 86)	20 (6 to 44)
Stress			
PSS-10 [22]	≥9	83 (81 to 88)	75 (72 to 81)
IES-R [22]	≥18	86 (67 to 94)	83 (76 to 88)
ERI [23]	≥2	73 (59 to 93)	89 (85 to 99)
PC-PTSD [24]	≥3	57 (45 to 68)	77 (67 to 82)
Anxiety			
GAD-2 [25]	≥3	73 (39 to 94)	83 (76 to 89)
GAD-7 [25]	≥10	64 (31 to 89)	86 (78 to 91)
OASIS [25]	≥8	82 (48 to 98)	82 (75 to 88)
HADS-A [25]	≥8	90 (85 to 93)	78 (69 to 85)
Depression			
BDI [26]	≥10	91 (86 to 96)	79 (52 to 100)
CES-D [26]	≥16	84 (79 to 89)	74 (68 to 80)
PRIME-MD [26]	≥1	91 (81 to 100)	66 (48 to 84)
GHQ [26]	≥4	86 (76 to 95)	66 (57 to 74)
HADS-D [26]	≥11	69 (45 to 86)	86 (73 to 93)
PHQ-9 [26]	≥10	88 (74 to 96)	88 (85 to 90)
Zung-SDS [26]	≥50	86 (73 to 100)	76 (57 to 95)
HSCL [26]	≥43	82 (71 to 93)	73 (52 to 94)

Abbreviations: BDI, Beck Depression Inventory; CES-D, Center for Epidemiological Studies Depression; ESS Epworth sleepiness score; ERI, Effort Reward Imbalance; GAD-2, 2-item Generalized Anxiety Disorder Scal; GAD-7, Generalized Anxiety Disorder 7-item; GHQ, Primary Care Evaluation of Mental Disorders; HADS-A, Hospital Anxiety and Depression Scale-Anxiety Subscale; HADS-D, Hospital Anxiety and Depression Scale-Depression Subscale; HSCL, Hopkins Symptom Checklist; IES-R, 22-item Impact of Event Scale–Revised; ISI, The seven-item Insomnia Severity Index; OASIS, Overall Anxiety Severity and Impairment Scale; PC-PTSD, 4-item Primary Care PTSD screen; PHQ-9, Patient Health Questionnaire-9; PRIME-MD, Primary Care Evaluation of Mental Disorders; PSQI, Hospital Anxiety and Depression Scale; PSS-10, 10-item Perceived Stress Scale; Zung-SDS, Zung Self-Rating Depression Scale.

eFigure 1. Assessment of Small Study Effects by Funnel Plot for the 16 Studies Reporting on Insomnia or Insomnia Symptoms Prevalence

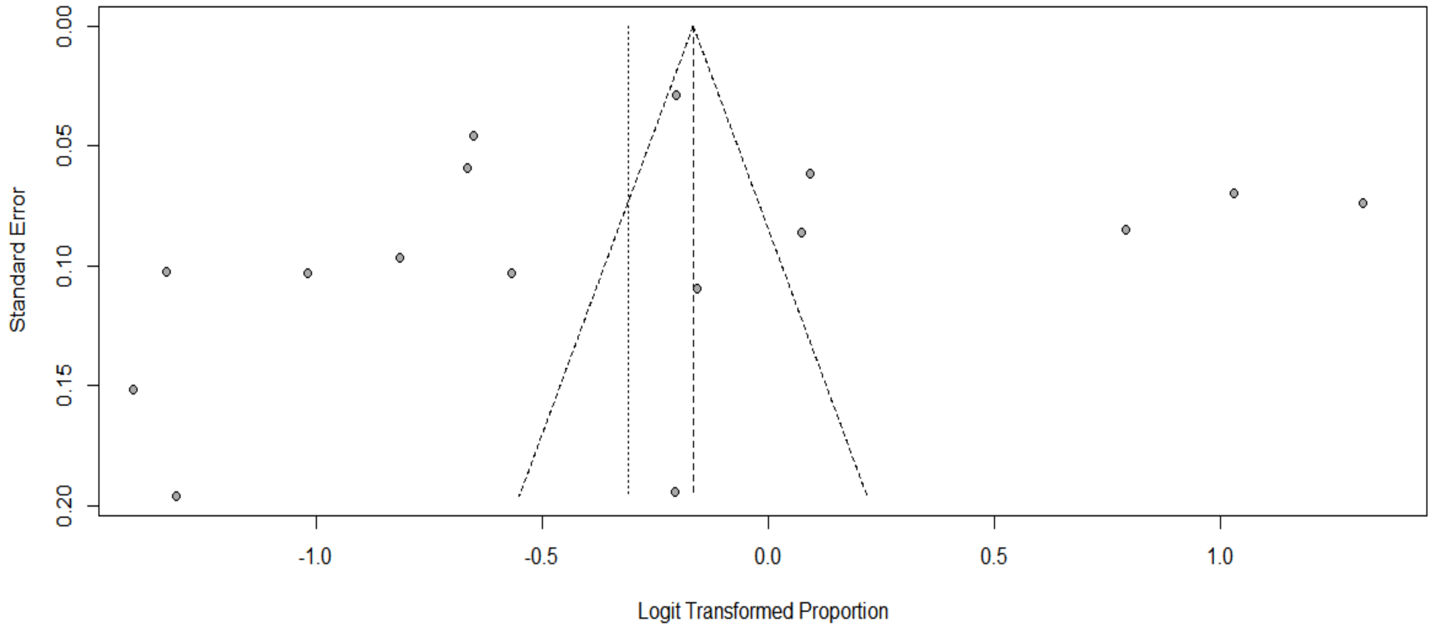

eFigure 2. Assessment of Small Study Effects by Funnel Plot for the 10 Studies Reporting on Stress or Stress Symptoms Prevalence

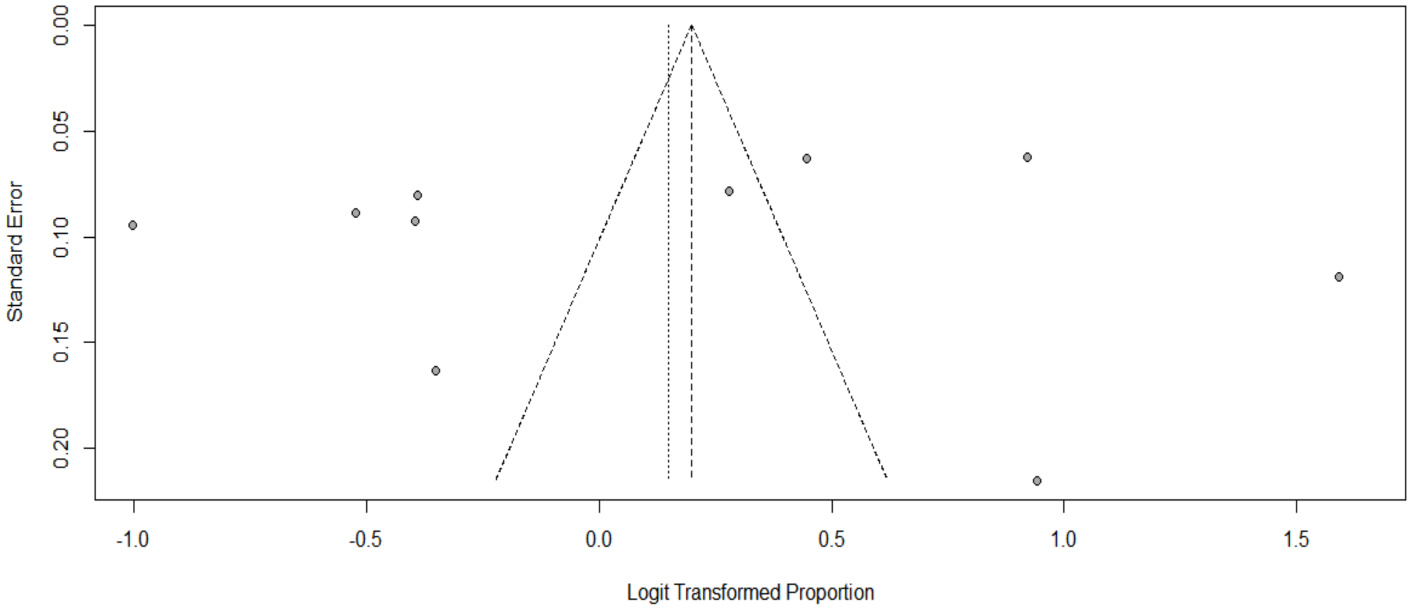

eFigure 3. Assessment of Small Study Effects by Funnel Plot for the 19 Studies Reporting on Anxiety or Anxiety Symptoms Prevalence

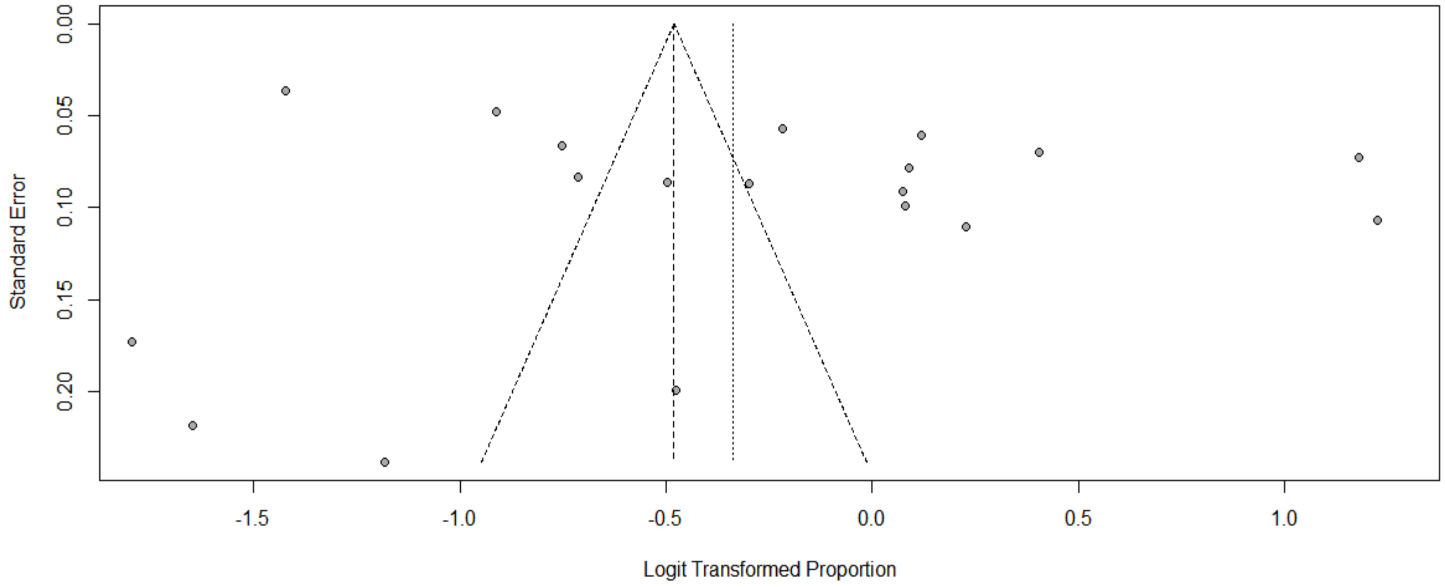

eFigure 4. Assessment of Small Study Effects by Funnel Plot for the 19 Studies Reporting on Depression or Depressive Symptoms Prevalence

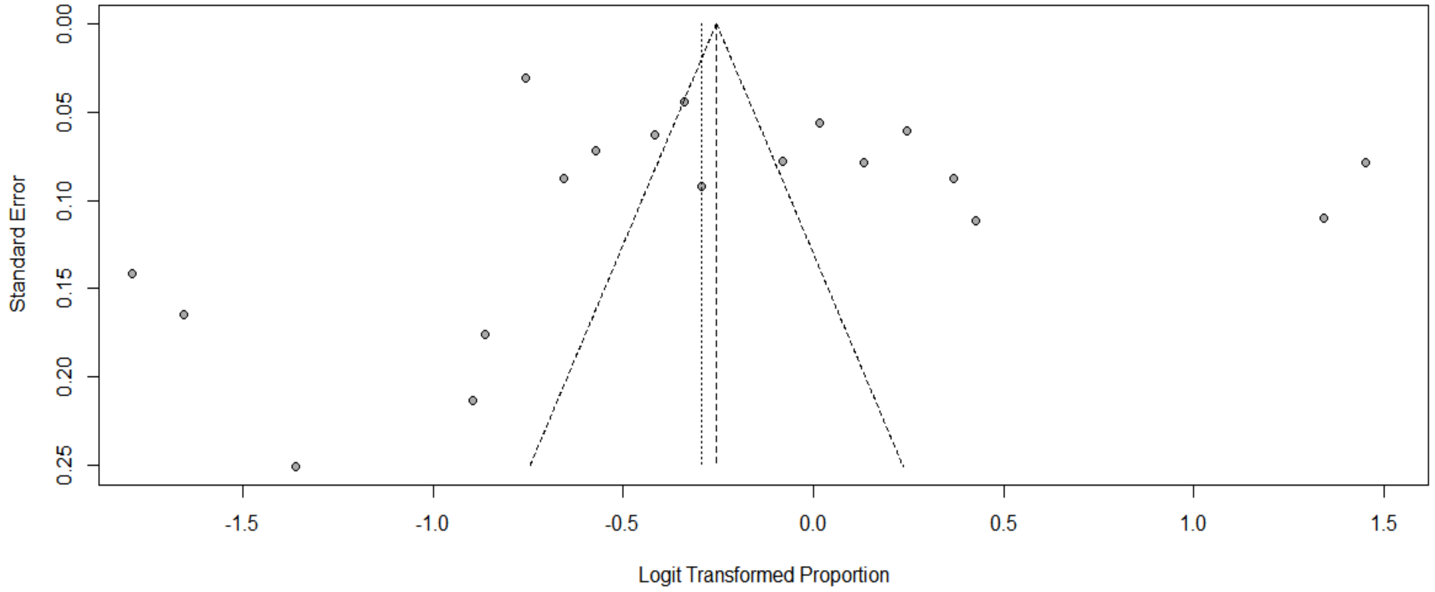

References

1. Almater AI, Tobaigy MF, Younis AS, et al. Effect of 2019 Coronavirus Pandemic on Ophthalmologists Practicing in Saudi Arabia: A Psychological Health Assessment. *Middle East Afr J Ophthalmol.* 2020(27):79-85. doi:10.4103/meajo.MEAJO_220_20

2. Alshekaili M, Hassan W, Al Said N, et al. Factors associated with mental health outcomes across healthcare settings in Oman during COVID-19: frontline versus non-frontline healthcare workers. *BMJ Open.* 2020(10):e042030. doi:10.1136/bmjopen-2020-042030

3. Cui Q, Cai Z, Li J, et al. The Psychological Pressures of Breast Cancer Patients During the COVID-19 Outbreak in China-A Comparison With Frontline Female Nurses. *Front Psychiatry.* 2020(11):559701. doi:10.3389/fpsyt.2020.559701

4. Elkholy H, Tawfik F, Ibrahim I, et al. Mental health of frontline healthcare workers exposed to COVID-19 in Egypt: A call for action. *Int J Soc Psychiatry.* 2021(67):522-31. doi:10.1177/0020764020960192

5. Giardino DL, Huck-Iriart C, Riddick M, et al. The endless quarantine: the impact of the COVID-19 outbreak on healthcare workers after three months of mandatory social isolation in Argentina. *Sleep Med.* 2020(76):16-25. doi:10.1016/j.sleep.2020.09.022

6. Haravuori H, Junttila K, Haapa T, et al. Personnel Well-Being in the Helsinki University Hospital during the COVID-19 Pandemic-A Prospective Cohort Study. *Int J Environ Res Public Health.* 2020(17) doi:10.3390/ijerph17217905

7. Lai J, Ma S, Wang Y, et al. Factors Associated With Mental Health Outcomes Among Health Care Workers Exposed to Coronavirus Disease 2019. *JAMA Netw Open.* 2020(3):e203976. doi:10.1001/jamanetworkopen.2020.3976

8. Liu T, Zheng Z, Sha X, et al. Psychological impact in non-infectious disease specialists who had direct contact with patients with COVID-19. *BJPsych Open.* 2020(7):e8. doi:10.1192/bjo.2020.147

9. Magnavita N, Soave PM, Ricciardi W, et al. Occupational Stress and Mental Health among Anesthetists during the COVID-19 Pandemic. *Int J Environ Res Public Health.* 2020(17) doi:10.3390/ijerph17218245

10. Shechter A, Diaz F, Moise N, et al. Psychological distress, coping behaviors, and preferences for support among New York healthcare workers during the COVID-19 pandemic. *Gen Hosp Psychiatry.* 2020(66):1-8. doi:10.1016/j.genhosppsych.2020.06.007

11. Tiete J, Guatteri M, Lachaux A, et al. Mental Health Outcomes in Healthcare Workers in COVID-19 and Non-COVID-19 Care Units: A Cross-Sectional Survey in Belgium. *Front Psychol.* 2020(11):612241. doi:10.3389/fpsyg.2020.612241

12. Wang LQ, Zhang M, Liu GM, et al. Psychological impact of coronavirus disease (2019) (COVID-19) epidemic on medical staff in different posts in China: A multicenter study. *J Psychiatr Res.* 2020(129):198-205. doi:10.1016/j.jpsychires.2020.07.008

13. Youssef N, Mostafa A, Ezzat R, et al. Mental health status of health-care professionals working in quarantine and non-quarantine Egyptian hospitals during the COVID-19 pandemic. *East Mediterr Health J.* 2020(26):1155-64. doi:10.26719/emhj.20.116

14. Azoulay E, Pochard F, Reignier J, et al. Symptoms of Mental Health Disorders in Critical Care Physicians Facing the Second COVID-19 Wave: A Cross-Sectional Study. *Chest.* 2021(160):944-55. doi:10.1016/j.chest.2021.05.023

15. Di Mattei VE, Perego G, Milano F, et al. The "Healthcare Workers' Wellbeing (Benessere Operatori)" Project: A Picture of the Mental Health Conditions of Italian Healthcare Workers during the First Wave of the COVID-19 Pandemic. *Int J Environ Res Public Health.* 2021(18) doi:10.3390/ijerph18105267

16. Fiol-DeRoque MA, Serrano-Ripoll MJ, Jiménez R, et al. A Mobile Phone-Based Intervention to Reduce Mental Health Problems in Health Care Workers During the COVID-19 Pandemic (PsyCovidApp): Randomized Controlled Trial. *JMIR Mhealth Uhealth.* 2021(9):e27039. doi:10.2196/27039

17. Guo WP, Min Q, Gu WW, et al. Prevalence of mental health problems in frontline healthcare workers after the first outbreak of COVID-19 in China: a cross-sectional study. *Health Qual Life Outcomes.* 2021(19):103. doi:10.1186/s12955-021-01743-7

18. Lee J, Lee HJ, Hong Y, et al. Risk Perception, Unhealthy Behavior, and Anxiety Due to Viral Epidemic Among Healthcare Workers: The Relationships With Depressive and Insomnia Symptoms During COVID-19. *Front Psychiatry.* 2021(12):615387. doi:10.3389/fpsyt.2021.615387

19. Wright HM, Griffin BJ, Shoji K, et al. Pandemic-related mental health risk among front line personnel. *J Psychiatr Res.* 2021(137):673-80. doi:10.1016/j.jpsychires.2020.10.045

20. Morin CM, Belleville G, Bélanger L, et al. The Insomnia Severity Index: psychometric indicators to detect insomnia cases and evaluate treatment response. *Sleep.* 2011(34):601-8. doi:10.1093/sleep/34.5.601

21. Alsaadi SM, McAuley JH, Hush JM, et al. Detecting insomnia in patients with low back pain: accuracy of four self-report sleep measures. *BMC Musculoskelet Disord.* 2013(14):196. doi:10.1186/1471-2474-14-196

22. Lee EH. Review of the psychometric evidence of the perceived stress scale. *Asian Nurs Res (Korean Soc Nurs Sci).* 2012(6):121-7. doi:10.1016/j.anr.2012.08.004

23. Siegrist J, Dragano N, Nyberg ST, et al. Validating abbreviated measures of effort-reward imbalance at work in European cohort studies: the IPD-Work consortium. *Int Arch Occup Environ Health.* 2014(87):249-56. doi:10.1007/s00420-013-0855-z

24. Li J, Zhang W, Chen W, et al. Applications of the Chinese version of the primary care PTSD screen for DSM-5 (PC-PTSD-5) for children. *J Affect Disord.* 2019(254):109-14. doi:10.1016/j.jad.2019.05.021

25. Hitchon CA, Zhang L, Peschken CA, et al. Validity and Reliability of Screening Measures for Depression and Anxiety Disorders in Rheumatoid Arthritis. *Arthritis Care Res (Hoboken).* 2020(72):1130-39. doi:10.1002/acr.24011

26. Williams JW, Jr., Pignone M, Ramirez G, et al. Identifying depression in primary care: a literature synthesis of case-finding instruments. *Gen Hosp Psychiatry.* 2002(24):225-37. doi:10.1016/s0163-8343(02)00195-0
